# Introduction of Materials Genome Technology and Its Applications in the Field of Biomedical Materials

**DOI:** 10.3390/ma16051906

**Published:** 2023-02-25

**Authors:** Yashi Qiu, Zhaoying Wu, Jiali Wang, Chao Zhang, Heye Zhang

**Affiliations:** School of Biomedical Engineering, Shenzhen Campus of Sun Yat-sen University, Shenzhen 518107, China

**Keywords:** biomedical material, material genome technology, database, high-throughput technology, algorithm model

## Abstract

Traditional research and development (R&D) on biomedical materials depends heavily on the trial and error process, thereby leading to huge economic and time burden. Most recently, materials genome technology (MGT) has been recognized as an effective approach to addressing this problem. In this paper, the basic concepts involved in the MGT are introduced, and the applications of MGT in the R&D of metallic, inorganic non-metallic, polymeric, and composite biomedical materials are summarized; in view of the existing limitations of MGT for R&D of biomedical materials, potential strategies are proposed on the establishment and management of material databases, the upgrading of high-throughput experimental technology, the construction of data mining prediction platforms, and the training of relevant materials talents. In the end, future trend of MGT for R&D of biomedical materials is proposed.

## 1. Introduction

As a dynamic and fast-growing branch of materials science, biomedical materials are used to diagnose and treat physiological diseases, repair or replace biological tissues or organs to enhance or restore their functions. Biomedical materials have been extensively applied in clinical practice in forms of various medical devices including sutures, scaffolds, dentures, artificial bones, and even artificial hearts, etc. The research and development (R&D) of innovative biomedical materials involves the breakthrough in a wide range of knowledge including materials science, engineering, medicine, and life science, etc.

The utilization of biomedical materials dates back to 3500 B.C. Since the first use of horsehair as suture, the fast-growing developments in life science and materials science have led to extensive applications of biomedical materials. However, the tissues or organs are extremely complex, the ability of biomedical materials to precisely regulate the growth, regeneration, and repair is far from satisfaction. The growing curiosity on the structure–property relationship of biomedical materials as well as the interactions between biomedical materials and cells/tissues/organs may lead to the investigation of these topics to foster the R&D of new biomedical materials. It is often achieved by development of novel chemical structure, combination of materials, or fusion of materials with living cells, etc.

Traditionally, most biomedical materials are developed via the trial-and-error method. In detail, different material components and synthetic techniques could be explored based on existing theories or experiences to achieve desired material properties. During this process, repeated experiments are usually required to validate the design or formulation, which would inevitably lead to the sacrifice and waste of material/time. Such trial-and-error approach may be effective at small scale, but would greatly hinder the innovation of materials and the development of related industries in the case of complex tasks or large scale. In terms of economic and time costs, there is an urgent need for a new method to overcome the drawbacks of traditional trial-and-error method in the R&D of biomedical materials [1,2,3,4].

Materials genome technology (MGT) is a superior tool of materials research to the traditional trial-and-error method. MGT utilizes high-throughput experimental technique while adding data management and computational tools. The data are analyzed using computational tools to explore potential links between material parameters and material properties. In this way, the ideal material can be discovered more efficiently. This can improve experimental efficiency, reduce costs, and perhaps reduce experimental errors.

This review presents the recent progress of the application of MGT in the R&D of biomedical materials to halve their development cycle and cost. Core concepts of biomedical materials and MGT are briefly introduced, followed by the application of MGT in the R&D of metallic, inorganic non-metallic, polymeric, and composite biomedical materials. Finally, future trends in the MGT empowered R&D of innovative biomedical materials are proposed.

## 2. Materials Genome

**Figure 1:**
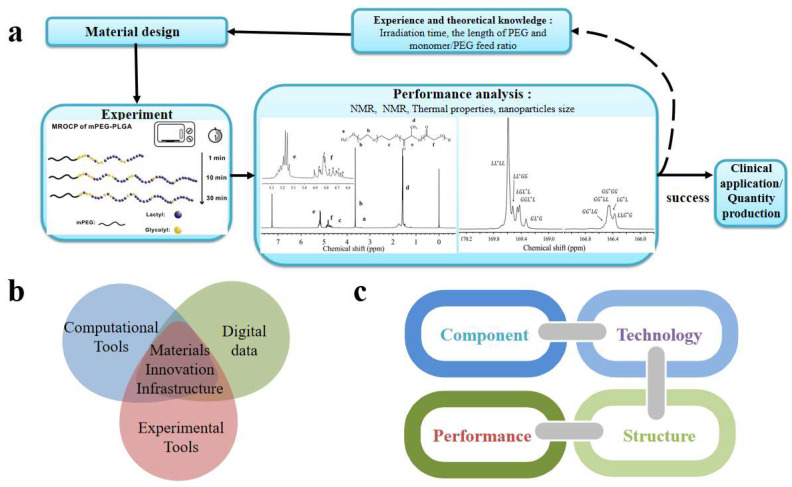
The traditional trial and error research process with Jing [9] as an example (**a**). Three elements of MGI (**b**) and the connection between material composition, synthesis technology, structure, and material performance (**c**).

As the first program on materials genomic study, the Materials Genome Initiative (MGI) was launched in 2011 and includes three major aspects covering database establishment, experimental techniques, and means of material calculation (Figure 1b) [5,6,7,8]. Through “simulation and prediction followed by experimental verification” approach, the MGI aims at establishing the link between composition, process, microstructure, and performance (Figure 1c) to facilitate the R&D of materials [5]. Such link could be subsequently used in the design and optimization of new materials to meet the demand for material performance. As a consequence, the MGT may also halve the development cycle of new materials as well as the development cost in industry, which is almost impossible through the traditional trial and error approach (Figure 1a).

Database: It is hard to find valid and high-quality data from the massive amount of data when data are the basis for materials research, and the creation of databases becomes even more important. Ideal material database should be able to store large amount of data, manage the data in a standardized way, and be retrieved and stored easily by users.

Famous material information databases have been established, such as Materials Project, AFLOW, and OQMD, etc. In China, efforts have been devoted for the establishment of various large-scale general material databases since the [10,11] data from different sources and categories are integrated to meet the varied needs of users such as non-ferrous metal database, alloy steel database, material corrosion database, and aviation material database (Table 1) [12,13,14].

High-throughput experimental tools: As the essential part of the MGT, high-throughput synthesis and characterization of materials refers to the preparation and characterization of samples with different structures or components in parallel and in large quantities in a relatively shorter time [15]. Usually, only very limited and inefficient data could be acquired through individual experiments with inevitable human errors. High-throughput experiments can help improve the accuracy and reproducibility of the data at higher efficiency, and consequently accelerate the establishment of material databases, testing the accuracy of theoretical models, and screening of new materials. Nowadays, high-throughput/combinatorial methods have been successfully applied to the development and production of metallic, ceramic, inorganic, and polymeric materials [16,17,18,19,20,21].

Materials calculation methods: Materials calculation methods usually refers to various types of materials computing software and algorithmic models. Facing the complex potential connections between material components, structures, processes, and properties, which usually cannot be discovered directly by researchers, the help of some material computational means is urgently required. Although high-throughput first-principles calculations and density functional theory have achieved appreciable success in predicting and optimizing new materials, the huge amount of calculation makes it impossible to obtain ideal results quickly when the structure is more complex or the material search space is larger [21]. Because of their ability of establishing accurate material performance prediction models from existing theoretical and empirical data, machine learning techniques have received great attention in predicting material properties [18,22,23], optimizing material composition [24,25], and discovering new materials [26].

## 3. Algorithmic Models in Material Genome Technologies

Because of the large volume of computational tasks and the automatic processing of computational results, it is extremely challenging to make full use of the huge and complex data and to reveal potential connections between relevant parameters and performance of materials. Computational tools, including various software and algorithm models, could be harnessed to solve the challenges in the processing of large amounts of data.

Regression, classification, or clustering tasks are usually employed in the processing of biomaterial data. Regression tasks mainly deal with continuous or real number, while classification or clustering tasks result in discrete outcome by inputting data with or without label. Both regression and classification aim at discovering the relationship between data points through a predictive model, and achieve reliable predicted outcome; for these purposes, some algorithms, such as support vector machines (SVM), random forests, and Bayesian neural networks, etc., can be used for both regression and classification tasks in case of biomedical materials data (Table 2).

**Table 2:** The introduction of some common algorithms.

	Task	The Algorithm	The Idea of Algorithm	Feature	Application
**3.1**	Regression	Back Propagation Neural Network (BPNN)	A multi-layer feedforward network trained according to the error back propagation algorithm, which continuously adjusts the weights and thresholds of the network through back propagation to minimize the sum of squared errors of the network.	It has nonlinear mapping capabilities, strong self-learning and self-adaptation capabilities, high generalization capabilities and fault tolerance; but the convergence speed is slow, and local minimization problems are prone to occur; high sample dependence	[27,28,29,30,31,32,33,34,35,36,37,38,39]
**3.2**	Regression & Classification	Radial Basis Function Neural Network (RBFNN)	RBF is used as the activation function of the hidden layer neurons, and the output layer is a linear combination of the output of the hidden layer neurons	The structure is simple, the training is concise, the learning convergence speed is fast, it can approximate any nonlinear function, and overcome the local minimum problem. Approximation ability, classification ability and learning speed are better than BPNN. But need more neurons	[33,40,41,42,43,44,45,46,47]
**3.3**	Classification	Probabilistic Neural Network (PNN)	It is a branch of RBFNN, which combines density function estimation and Bayesian decision theory on the basis of RBF network	The fault tolerance is good, the classification result is not sensitive to the choice of radial basis function, the number of neurons in each layer is fixed, and there is no need to retrain when the sample changes. But every sample has to be calculated and stored.	[48,49,50,51,52,53]
**3.4**	Regression & Classification	Support Vector Machine & Support Vector Regression (SVM&SVR)	Establish the maximum separation line or hyperplane for sample classification, and find a balance between model learning accuracy and learning ability to obtain the best promotion ability.	It can solve nonlinear problems with high precision and good generalization ability; it is difficult to implement large-scale training samples and it is difficult to solve multi-classification problems.	[20,33,54,55,56,57,58,59,60,61,62,63,64,65]
**3.5**	Regression & Classification	Random forest	Create multiple decision trees in a random manner. Different trees have different prediction results for the same sample. Combining these results, the final result is the average of all decision tree results.	For unbalanced data sets, errors can be balanced, high-dimensional data can be processed, and with good accuracy; but in the face of small samples, good classification results may not be obtained, and it is easy to overrun in some noisy regression problems combine.	[20,66,67,68,69,70,71,72,73,74]
**3.6**	Clustering	K-means Clustering	Use distance as the similarity evaluation index to cluster the samples with high similarity into a cluster	The algorithm is simple and fast, with strong interpretability, good clustering effect, difficult to determine parameters, sensitive to noise and abnormal points, poor clustering effect on severely unbalanced data, and it takes a long time to process large sample sizes.	[75,76,77,78,79,80,81]
**3.7**	Regression & Classification	Convolutional Neural Network (CNN)	Multi-layer representation of the target using convolution and multi-layer network structure. It is expected that the abstract information contained in the data can be expressed through the multi-layer high-level features to obtain better feature robustness	Local connection and weight-sharing greatly reduce the number of parameters, and there is no pressure on high-dimensional data processing, reducing the risk of overfitting, no need to manually select features, no complicated preprocessing process when processing image data, but when adjusting parameters, a large sample size is required, GPU is best for training, and the physical meaning is not clear	[82,83,84,85,86,87,88]

### 3.1. Back Propagation Neural Network

Back propagation neural network (BPNN) [89] is characterized by two processes: forward propagation and error back propagation. In forward propagation, data are processed layer by layer, and the error was evaluated between the result of the output layer and that of the actual sample. In back propagation, the calculated error is back propagated, and then the weight and threshold of the network were continuously adjusted to minimize the error sum of squares of the network. Because of its good nonlinear mapping ability and strong self-learning and self-adaptive ability, BPNN has become one of the most widely used neural networks at present. However, in the case of a complex target task, the network converges slowly and easily to a local minimum, and the global optimal result cannot be obtained.

### 3.2. Radial Basis Function Neural Network

Radial basis function neural network (RBFNN) is a single hidden layer, function approximation-based feedforward neural network [90]. After selecting the radial basis function such as Gaussian function and multiquadric function, the output is obtained according to the distance between the sample and the center point. Compared with BPNN, RBFNN is structurally simpler, exhibits higher convergence speed, and rarely produces local optimum. In addition, RBFNN not only has powerful nonlinear approximation capability which transforms linearly indistinguishable problems into linearly divisible ones, but also can be applied to data classification problems. However, it is difficult to determine the center point of the hidden layer, the width of the path base, as well as and the number of nodes, which may have a substantial impact on the output.

### 3.3. Probabilistic Neural Network

Probabilistic neural networks (PNN) is a simple structured neural network based on Bayesian decision theory, which is often used for pattern classification; PNN can also be regarded as a branch of RBFNN, it combines density function estimation and Bayesian decision theory [91]. The unique advantage of PNN is that it is not necessary to retrain the network when adding or reducing samples, and the classification results are insensitive to the choice of radial basis functions. In addition, the number of neurons in each layer of the network is relatively fixed, which is easy to implement in hardware. The drawback of PNN lies in the high complexity in computation and space of the network; in addition, individual computation and storage are required for each sample.

### 3.4. Support Vector Machine and Support Vector Regression

SVM is a binary classification model, which aims to find a line or plane with the largest geometric interval to classify the samples [92]. In case of nonlinear problems, the vector can be mapped to a higher dimensional space to find the best plane to classify sample. Support vector regression (SVR) is an application of SVM to regression problems [93]. It finds a plane to fit all the sample data so that the total variance of the sample distances from the plane is minimized, instead of separating the sample points. Both of them are powerful models that harness relatively fewer samples to find a balance between learning accuracy and learning ability, and obtain the best generalization ability. However, there are still some difficulties when dealing with multi-classification problems and large-scale samples, and their results are sensitive to the choice of kernel function.

### 3.5. Random Forest

Random forest (RF) was proposed as a machine learning algorithm for classification and regression [94]. The “forest” means that the RF algorithm is a combination of multiple decision trees, and “random” means that when training each tree, a subset is randomly selected for training, and the remaining is used for error evaluation. RF has appreciable accuracy even when applied to large data sample sets and missing data sets. However, when dealing with small data sets or low-dimensional data sets, RF may not produce good classification results. In addition, overfitting is likely to occur when processing some noisy data.

### 3.6. K-Means Clustering

For some unlabeled sample data, only the similarity between the data can be used to group the data. For example, the unlabeled sample data with high similarity form a cluster, which is called clustering [95]. K-means clustering is one of the most classical clustering algorithms. Given the number of clusters, the initial cluster centroids are randomly set up. The spatial distance is used as the evaluation index of similarity, so that the sample points within a cluster are as close as possible, and the sample points of different clusters are as far away as possible. Such an algorithm is simple, explanatory, and effective, and therefore is widely used. It was also found that the number of clusters has a great influence on the clustering results, as well as there is no reference and a lot of trials and experience are needed. The algorithm is sensitive to outliers and noise, and it is also difficult to obtain good clustering results for severely imbalanced samples.

### 3.7. Convolutional Neural Network

Inspired by Hubel’s research on cat visual cortex cells, the convolutional neural network (CNN) was proposed [96]. The combination of convolutional and pooling layers of CNN can automatically perform feature extraction, and use local connectivity and weight-sharing to greatly reduce the number of the model parameters, reduce the risk of overfitting, and also simplify the complexity of the model. This is what distinguishes CNN from other neural networks. This advantage is even more evident when processing speech, image, or video data.

Although CNN does not require manual feature selection, which reduces human intervention, the question on what features is automatically extracted remains unanswered for the time being. In addition, CNN requires a large number of samples for model tuning, and usually requires GPU for model training. The puzzling functions and uncertain working principle of CNN have been questioned, but the performance of convolutional neural networks has been greatly improved over other methods.

The above-mentioned algorithm models have been widely used in processing biomedical data, and improvements have been extensively proposed.

## 4. Applications of MGT in the R&D of Biomedical Materials

### 4.1. Metallic Materials

Metals, especially alloys, have excellent mechanical properties, fatigue resistance, processability, and appreciable biocompatibility, and are widely used in the fabrication of implantable medical devices for the treatment of orthopedics, dentistry, and cardiovascular diseases. However, metallic materials are susceptible to the physiological environment and may lead to a series of problems such as degradation/corrosion, toxicity, and fatigue failure [97,98,99,100].

**Figure 2:**
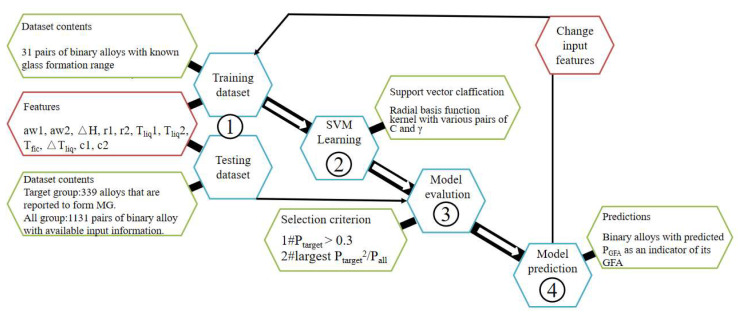
Overall setup for SVM modeling. Reprinted (adapted) with permission from SUN [54]. Copyright 2017 American Chemical Society.

Amorphous alloys possess good strength, hardness, wear resistance, corrosion, and soft magnetic properties, which are not available in traditional alloys and therefore have broad biomedical applications [101,102]. Although empirical guidelines have been constructive in the design of amorphous alloys, such approaches are characteristically time-consuming and reckless to some extent [103]. The intervention of artificial intelligence can not only improve the R&D efficiency, but also explore the unknown parameter space. With the aid of MG technology, the relationship between the resistivity and glass-forming ability (GFA) of amorphous Ir-Ni-Ta—(B) alloys was explored via high-thought characterization of resistance and components. A set of development methods of high-thought amorphous materials was built including the preparation of composite films, rapid characterization of composition, structure, and glass-forming ability, and a class of high-temperature amorphous materials was successfully designed [104]. Through SVM classification, the prediction of the GFA of binary alloys with random composition was achieved, and the prediction efficiency was also improved via using a larger database and changing the input descriptors (Figure 2) [54]. In addition, to better understand and predict the GFA of new alloys, machine learning clustering technique was harnessed to learn the structural properties of metallic glasses [105].

Pure titanium metal is known for not only its superior mechanical performances but also the reactivity under certain biochemical environment, thus it is not suitable for the fabrication of implantable medical devices; titanium alloys with improved resistance to corrosion and better biocompatibility could be a substitution to the pure titanium metal in the manufacturing of clinical implant devices. The participation of artificial intelligence is helpful to the innovation of titanium alloy [106,107,108]. Banerjee collected the indentation hardness and elastic modulus of titanium alloy samples to build a database of composition, microstructure, and mechanical properties, which became the basis of a fuzzy logic-based neural network building and predicting; the predicted results were subsequently validated through experiments [109]. Wan constructed BPNN to predict the high-temperature rheological stresses of Ti-2.7Cu alloy and provide theoretical support for practical hot forming of the alloy [110]. Based on the PNN and databases from experimental research on titanium alloys, Kulyk created a software to define the optimal microstructure and properties of titanium alloy products [48]. Tkachenko described a method for identifying material categories using second-order Kolmogorov–Gabor polynomials and RF algorithms; this method was then used to determine the basic properties and identify the category of the alloy of a material based on parameters such as microstructure and elemental composition of a titanium alloy powder. This approach can be used to optimize the development of powdered materials [66]. Izonin also used Ito decomposition and logistic regression to classify alloys in order to select materials with appropriate properties to design biocompatible medical products. [111]. Izonin combined Wiener polynomials and SVM to classify medical titanium alloy implant materials, this combined method exhibited higher accuracy as well as shorter training time [55]. The above studies have clearly demonstrated the efficacy of different algorithms in guiding the optimization of microstructure and processing routes of titanium alloys.

**Figure 3:**
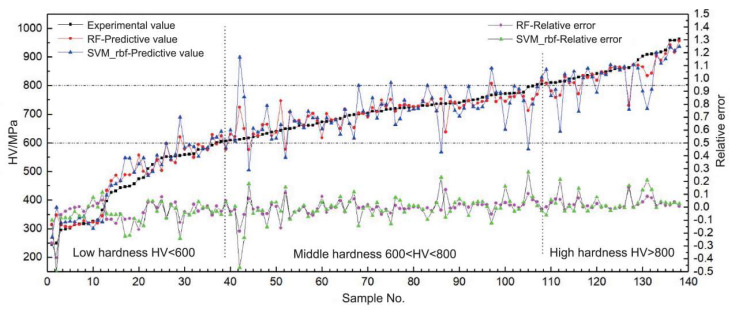
Experimental values, predictive values, and relative errors of predictive values to the hardness of 138 alloy samples. Reprinted (adapted) with permission from WANG [20]. Copyright 2020 Materials China.

High entropy alloy (HEA), as a rapidly developing new metallic material, is a class of alloy with high strength, wear, and corrosion resistance, and may have wide clinical application [112]. However, the composition of high-entropy alloys is complex; and there is no linear relationship between performance and entropy value, so it is impossible to design multi-component materials with excellent performance merely by entropy of mixing. In addition, the number of constituent elements of the alloy gradually increases, and the cost of the alloy also rises accordingly. Through the combination of high-throughput experimental techniques with artificial intelligence algorithms, the experimental efficiency could be enhanced and experimental compositions could also be explored to a wider space. Moorehead explored the composition space of the HEA through high-throughput synthesis and characterization combined with modeling techniques, and the development of alloys and assess of the relative stability thereof could be significantly accelerated [113]. Coury utilized high-throughput nano-indentation techniques to effectively predict the yield strength and hardness trends of HEA, the number of experiments required to find compositions in a large composition space was greatly reduced, thus promoting the development of multicomponent alloys [19]. Liu prepared 138 alloy samples through full-flow high-throughput preparation of alloys, and then constructed predictive models using different machine learning algorithms. The newly proposed method was at least 20 times faster than that of a permutation-based search in the full-component space (Figure 3) [20].

It is important to distinguish the phases of high-entropy alloys for material design. Ouyang optimized feature variables and used SVM model for phase distinction. It was found that the difference in elastic energy and atomic size had a significant effect on the formation of different phases. Importantly, machine learning (especially the SVM combined KPCA) showed its powerful role in the prediction of alloy phases [65].

### 4.2. Polymeric Materials

The demand of polymeric biomaterials, either natural or synthetic, has become increasingly urgent in recent years. Most polymeric materials including polyethylene, poly(methyl methacrylate), silicone rubber, cellulose, gelatin, and chitosan are known for their good biocompatibility. However, most of these polymers suffer from insufficient mechanical strength and mismatch between material degradation and tissue regeneration [3,114,115].

Chitosan nanoparticles have been widely used as drug delivery matrix due to their unique biocompatibility, degradability, and antimicrobial activity. Amani analyzed the effect of four parametric variables in preparation of chitosan nanoparticles on the nanoparticle size, drug loading, and cytotoxicity using artificial neural networks, and ranked the influential degree of the variables on the dependent variable and optimized the nanoparticles [116]. In another work, Amani analyzed the effects of time and amplitude of ultrasonication on the size of nanoparticle during the preparation [23].

Alexander and co-workers investigated the preparation drug release matrix through 3D printing of 253 ink formulations in a high-throughput manner, and the functional properties including the release of paroxetine, cytotoxicity, printability as well as mechanical properties are screened [117].

After implantation of biomedical materials, the adsorption of protein and the attachment of cells are the main determinants of the applicability of medical implant materials, especially those applications involving tissue regeneration. Both the surface chemistry and physics of polymeric implant could affect the protein adsorption; in this term, the surface chemistry and physics of polymers are complex and a fingerprint profile could be developed as a characteristic representation of polymers in order to enable a reasonable discovery of new materials for specific applications. Machine learning models can be then trained to quickly predict the properties of new polymer formulations and provide uncertainty in the predictions [118]. In addition to study the pathogen infection of the implant, bacterial cell adhesion on the surface of implant was investigated; machine learning was utilized to quantitatively predict and screen the polymer surface adhesion, and the screened polymers can be candidates for implants or indwelling medical devices [119].

Poly(lactic acid)/ploylactide (PLA)-based composites are ideal materials in bone repair, but PLA suffers from low cell adhesion on the material surface, poor mechanical properties, and high cost, which greatly limit its clinical applications [120]. Rojek investigated the customized fabrication of a PLA hand exoskeleton using 3D printing technique, and the artificial neural network (ANN) optimization method supported by GA was used to calculate and optimize the process parameters and material selection to achieve the maximum tensile force of the hand exoskeleton component. The combination of AI and 3D printing not only can optimize PLA properties but also has provide a good inspiration for using artificial intelligence to customize patient solutions [39].

### 4.3. Inorganic Materials

Inorganic materials are known for their high melting point, hardness, and resistance to oxidation as well as potential biocompatibility in clinical use. The dissolution of metal ions from metallic implant may cause toxicity to host tissue, oxide films on the surface of metallic implant could be used to address this issue; it was found that both the formation and the thickness of oxide films may significantly affect the surface properties and biocompatibility of metallic implant, but the effect of process parameters on the film thickness is far from being elucidated and the general linear fitting methods cannot meet the needs in modeling such processes. To visually examine the quality of oxide film on the surface of magnesium alloy, Yang used genetic algorithm (GA) to optimize the initial weights and thresholds of the BPNN to construct a film thickness prediction model (GA-BP). The GA-BP model was found to have better prediction accuracy than the BPNN model [121].

Titanium dioxide (TiO_2_) nanotube arrays have been found to promote cell adhesion, proliferation, and differentiation, and can strongly bind to titanium substrate; such characteristics of titanium dioxide nanotube arrays could be harnessed to improve the biocompatibility of titanium/titanium alloy implants and have attracted the attention of biomaterial researchers. Mou fabricated gradient TiO_2_ nanotubes micro-patterned films on the surface of titanium to facilitate the high-throughput screening of protein adsorption, platelet adhesion, bacterial adhesion, and the effect of octacalcium phosphate membrane layer construction. The gradient TiO_2_ nanotube micropatterning proved to be an effective tool in high-throughput screening for biomedical applications [122].

Besides the oxide films on metal surfaces, metal oxides are also good options for biomaterials. In order to select suitable metal oxides quickly and accurately, Hu applied machine learning and feature selection to predict the physical properties of metal oxides, and found that the RFR model combining different feature selection methods (Variance Threshold, Univariate feature selection, and Least absolute shrinkage and selection operator) achieved better results in terms of prediction accuracy [74].

### 4.4. Composite Materials

Composites are materials made by combining two or more materials with different properties in order to effectively make up for the deficiencies in biological and physicochemical properties of a single material, and further improve the applicability of the material in clinical applications [120,123,124].

**Figure 4:**
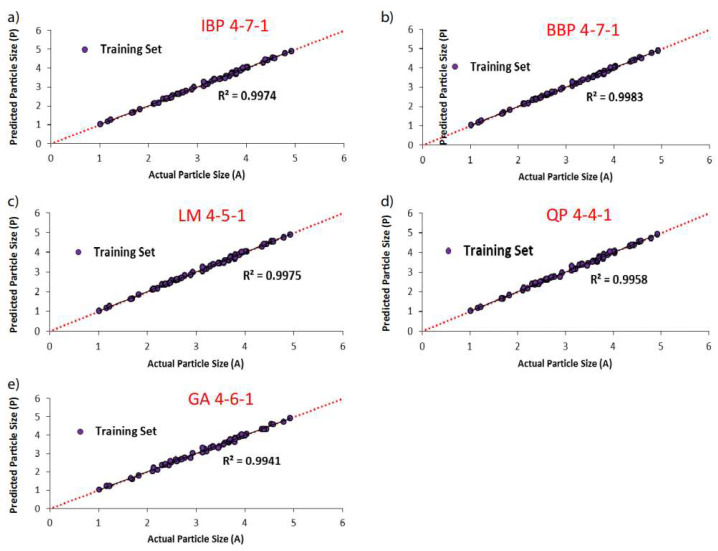
Scatter plots of the predicted Dp (in nm) against an actual range of Dp for the training set of data for the selected topologies with Soroush SOLTANI [38]. The designated topologies were 4-7-1, 4-7-1, 4-5-1, 4-4-3, and 4-6-1 for (**a**) IBP; (**b**) BBP; (**c**) LM; (**d**) QP; (**e**) GA algorithms, respectively Reprinted (adapted) with permission. Copyright 2020.

Nano-emulsions have been utilized as the carrier for oral drugs, but the cytotoxicity and low stability hinder their wide application. The artificial neural network analysis revealed that the concentration of surfactant is the main determinant of stability without causing dose-dependent cytotoxicity. Such findings paved the way for the preparation of nano-emulsions with optimized cytotoxicity and stability [125]. The nature of the composites can have an impact on the drug loading and the release behavior of the loaded drug. Bikiaris prepared a series of poly(ε-caprolactone)/poly(propylene glutarate) (PCL/PPGlu) polymer blends at different weight ratios as the matrix using risperidone as the model drug, followed by the evaluation of the interaction between the polymer and the drug. Artificial neural network, applied for simulation of the drug dissolution behavior, revealed a higher fitting and correlation compared with multiple linear regression (Figure 4) [37].

To better predict and optimize the performance of the carrier, the neural network using other algorithms have been explored. Varshosaz combined genetic algorithm and artificial neural network to optimize and simulate the synthetic process of agar nanosphere from agar, calcium chloride, hydroypropyl-β-cyclodextrin, and bupropione hydrochloride. Satisfactory consistence was achieved between the predicted and actual values of the ANN model [126]. Wu and co-workers found that the combination of neural network and genetic algorithm could better predict and optimize the formulation of nanoparticles than the response surface method to achieve better controlled release behavior [127].

Composite materials based on chitosan has good biocompatibility and biodegradability, so the composite material with chitosan as one of the components surpasses the traditional materials to a certain extent for potential clinical use [128,129]. Fourier transform infrared spectroscopy and differential scanning calorimetry were employed to investigate the interactions of variable chitosan and sodium tripolyphosphate in the formation of nanoparticles, artificial neural network was built based on these data and used to predict not only the particle size, but also the yield of nanoparticles [36]. Shang used the relevant data of fish skin collagen extraction process to establish a BPNN to analyze and study the different factors and levels in the extraction process, and screen the best parameters. Finally, the relative error between the predicted value obtained by the network and the actual value obtained by the orthogonal experiment is not more than 5%, which shows the feasibility of BP neural network combined with the orthogonal experiment to optimize the collagen extraction process, and the model has reliable predictive performance [130].

## 5. Perspectives and Outlook

Materials genome technologies have changed the traditional material R&D paradigm; however, tremendous efforts on the following topics are required to meet the fast-growing needs and challenges in the R&D of biomedical materials. Mapping the relationship between the components, structures, and properties of biomedical materials is complex and challenging. This challenge comes mainly from the difficulty in obtaining adequate data of biomedical materials, which are generally small sample data sets. In this regards, either the development of high-throughput experimental tools or data enhancement would be highly desirable [131,132]. In addition, image data of biomedical materials are underutilized; related tools for image feature extraction can be harnessed in image analysis of biomedical materials to help extract image information [133].

### 5.1. Establishment and Management of the Database

As an important foundation in the data-driven material R&D, the present biomedical material data are characterized by different sources, diverse types, and complexity, which to some extent hinder the rapid development of the perfect material data standard system and the establishment of data import template are prerequisite to deal with a variety of data types and formats as well as the systematic management and storage of data. My SQL database, which is small in size and low in cost, is a good option for the establishment of open material database. Artificial intelligence can also be used to automatically collect and classify the latest literature data. In addition, the experimental parameters have a huge impact on the performance and structure of the material. Relevant experimental conditions of the data should be supplemented and refreshed in the database to validate the simulation results.

In addition, the parameters in animal experiments should also be considered. This is unique and critical data for biomedical materials.

### 5.2. Development of High-Throughput Technology

Large-scale automation of the experiment and calculation process may significantly accelerate the establishment of the database. At this point, high-throughput techniques can be used to synthesize and characterize materials with high efficiency and precision. The use of high-throughput equipment is of great significance. It may not only solve the defects of manual experiments but also can strengthen the combination of high-throughput equipment with databases and calculation methods, and consequently improve the level of material development, production, and application. In addition, biomedical materials with nanometers and microns in size also require a significant reduction in experimental error.

### 5.3. Innovation of Algorithm

The simulation and prediction ability of algorithm model is essentially important for the R&D of biomedical materials. Animal models are commonly used in the pre-clinical investigation of biomedical material. However, the inevitable inconsistency between the physiological/pathological environments of the animals and humans would lead to confused results during the clinical translation. New algorithm models are needed to simulate as much as possible the real human body environment and the situation of biomedical materials in physiological tissue/organ. In addition, the relatively smaller data set derived from open databases or high-throughput synthesis/screening of biomedical materials does not meet the requirement of general machine learning and deep learning. It would be highly necessary to develop algorithms suitable for small data sets to obtain material data analysis results with higher accuracy.

### 5.4. Industrial Involvements

The majority of current efforts on the MGT for biomedical materials have been devoted to the basic research; however, the ultimate outcome or performance of biomedical materials strongly depend on the processing and manufacturing parameters, and the involvement of industry is far from satisfactory. One would foresee that the MGT may add emphasis to its application in R&D of biomedical materials by receiving more input from the industry.

## 6. Concluding Remarks

MGT has strongly accelerated the R&D of materials, including predicting rapidly and screening materials, optimizing the properties of biomedical materials. Meanwhile, the application of MGT in biomedical materials has also promoted the innovation and development of science and industrial technology, basic theories, key technologies, and equipment. In the future, the development opportunities of MGT may be harnessed to facilitate the R&D of biomedical material, and work out the bottlenecks and difficulties in related fields.

## Figures and Tables

**Table 1:** List of the large databases.

Institute	Dataset	Content
Duke University	AFLOW (http://www.aflowlib.org/, accessed on 24 February 2023)	Band structures, Bader charges, elastic properties, thermal properties, binary systems, binary entries, ternary systems, ternary systems, ternary entries, quaternary systems, quaternary entries
Northwestern University	OQMD (http://oqmd.org/, accessed on 24 February 2023)	DFT calculated thermodynamic and structural properties of materials
University of California at Berkeley	Materials Project (https://materialsproject.org/, accessed on 24 February 2023 )	Inorganic compounds, band structures, molecules, nano-porous materials, elastic tensors, piezoelectric tensors, intercalation electrodes, conversion electrodes
A group of engineers	MatWeb (http://www.matweb.com/, accessed on 24 February 2023)	Thermoplastic and thermoset polymers, metals, and other engineering materials.
Key to Metals AG	Total Materia (https://www.totalmateria.com/, accessed on 24 February 2023)	Metal, polymer, silicate, composite materials
National Institute for Materials Science (NIMS)	MatNavi (https://mits.nims.go.jp/, accessed on 24 February 2023)	Polymer database, Inorganic Material database, Metallic Material database, and Computational Electronic Structure database
Stahlschlüssel	Key to Steel (http://www.keytosteel.com/, accessed on 24 February 2023)	Structural and constructional steels, Tool steels, Valve steels, High temperature steels and alloys, Non-magnetizable steels, Heat-resisting steels, Heat conducting alloys, Stainless steels, Stainless steel castings, Welding filler materials
ASM International	ASM Online Databases (https://www.asminternational.org/materials-resources/online-databases, accessed on 24 February 2023)	Alloy Center Database, Alloy Phase Diagram Database, Failure Analysis Database, Heat Treater’s Guide Online, Medical Materials Database, Micrograph Database Pearson’s Crystal Data
Technical University of Denmark	Computational Materials Repository (https://cmr.fysik.dtu.dk, accessed on 24 February 2023)	Computational 2D Materials Database, ABSe3 materials, A2BCX4 materials, Ag-Au nanoparticles, PV and PEC materials, ABS3 materials, ABX2 materials, Monolayer transition metal dichalcogenides and -oxides, Perovskites, Porphyrin based dyes, New Light Harvesting Materials, CatApp database et al.
Springer Nature	Springer Materials (https://materials.springer.com/, accessed on 24 February 2023)	Metal, alloys, ceramics, semiconductors, polymers, and many more material types
Uppsala University	The Electronic Structure Project (http://gurka.fysik.uu.se/ESP/, accessed on 24 February 2023)	Electronic structure related information for inorganic compounds
Fritz Haber Institute	Novel Materials Discovery (https://nomad-lab.eu/, accessed on 24 February 2023)	Metal, nonmetal, conductor, ferromagnet, antiferromagnet, diamagnetic, semimetal, paili paramagnet, intermediate valence, luminescent, ferroelectric, ferrimagnet, intercalation compound, spin, thermoelectric, birefringent
FIZ Karlsruhe	ICSD (https://icsd.products.fiz-karlsruhe.de/, accessed on 24 February 2023)	Crystal structure data includes unit cell, space group, complete atomic parameters, site occupation factors, Wyckoff sequence, molecular formula and weight, ANX formula, mineral group, etc.
U.S. Department of Commerce	NIST (https://data.nist.gov/sdp/#/, accessed on 24 February 2023)	Crystal structure data of nonorganic compounds (including inorganics, ceramics, minerals, pure elements, metals, and intermetallic systems)
Chinese Academy of Sciences	Data Cloud of CAS (http://www.csdb.cn/, accessed on 24 February 2023)	Covering physics, chemistry, astronomy and space, materials, biology, geosciences, resources, environment, energy, ocean, and many other disciplines
Institute of Metal Research, Chinese Academy of Sciences	Material Science Database (http://www.matsci.csdb.cn/, accessed on 24 February 2023)	Metal materials, inorganic nonmetal materials, scintillation materials, silicon carbide materials, nano materials, organic polymer materials
Beijing University of science and technology	Materials Scientific Data Sharing Network (http://matsec.ustb.edu.cn/, accessed on 24 February 2023)	Material foundation, non-ferrous materials and special alloys, ferrous materials, composite materials, organic polymer materials, inorganic non-metallic materials, information materials, energy materials, biomedical materials, natural materials and products, building materials and road traffic materials
Beijing University of science and technology	Materials Genome Engineering Databases (http://www.mgedata.cn/, accessed on 24 February 2023)	First principles calculation database, material thermodynamics/kinetics data, energy materials, catalytic materials, special alloys, rare earth functional materials, biomedical materials, composite materials, hydrogen embrittlement and stress corrosion, superconducting materials, ferrous materials, etc.
National Center for Nanoscience and Technology	Nano Research Scientific Database (http://www.nano.csdb.cn/, accessed on 24 February 2023)	Carbon nanotubes, graphene, magnetic nanoparticles, etc.
Beijing University of science and technology	National Materials Corrosion and Protection Data Center (http://www.corrdata.org.cn/, accessed on 24 February 2023)	Ferrous metals, non-ferrous metals, building materials, coating materials and polymer materials, and more than 600 kinds of materials.
Institute of Chemistry, Chinese Academy of Sciences	Polymer Materials Database (http://polynavi.iccas.ac.cn/, accessed on 24 February 2023)	Contains data resources related to plastics, rubber, fiber, organic coatings, adhesives, and polymer additives
China Iron and Steel Research Institute	Asteel (http://www.atsteel.com.cn/, accessed on 24 February 2023)	All kinds of steel and welding materials

## Data Availability

Not applicable.

## References

[1] Liu J., Liu J., Attarilar S., Wang C., Tamaddon M., Yang C., Xie K., Yao J., Wang L., Liu C. (2020). Nano-modified titanium implant materials: A way toward improved antibacterial properties. Front. Bioeng. Biotechnol..

[2] Checchetto R., Rigotti D., Pegoretti A., Miotello A. (2019). Chloroform desorption from poly(lactic acid) nanocomposites: A thermal desorption spectroscopy study. Pure Appl. Chem..

[3] Winkeljann B., Bauer M.G., Marczynski M., Rauh T., Sieber S.A., Lieleg O. (2020). Covalent Mucin Coatings Form Stable Anti-Biofouling Layers on a Broad Range of Medical Polymer Materials. Adv. Mater. Interfaces.

[4] Lee T.-H., Kim S.-W., Ito Y., Son T.-I. (2019). Preparation of injectable forms of immobilized protein drugs using uv-curable gelatin derivatives. J. Ind. Eng. Chem..

[5] (2012). Executive Office of the President President's Council of Advisors on Science and Technology. Report to the President on Ensuring American Leadership in Advanced Manufacturing. https://obamawhitehouse.archives.gov/sites/default/files/microsites/ostp/pcast-advanced-manufacturing-june2011.pdf.

[6] Changwon S., Clyde F., James A.W., Edward O.P.-K. (2020). Evolving the materials genome: How machine learning is fueling the next generation of materials discovery. Annu. Rev. Mater. Res..

[7] Ritchie A. (2020). The impact of creating the next-generation materials genome initiative workforce. JOM.

[8] de Pablo J.J., Jones B., Kovacs C.L., Ozolins V., Ramirez A.P. (2014). The materials genome initiative, the interplay of experiment, theory and computation. Curr. Opin. Solid State Mater. Sci..

[9] Jing Y., Yang M., Dai S., Quan C., Liu J., Jiang Q., Zhang C., Liu B. (2018). Microwaves promote transesterification in the rapid synthesis of methoxy-poly(ethylene glycol)-block-poly(l-lactide-random-glycolide). Polymer.

[10] Marinova S., Deetman S., van der Voet E., Daioglou V. (2020). Global construction materials database and stock analysis of residential buildings between 1970–2050. J. Clean. Prod..

[11] Zhang X.-L., Pan J., Jin X., Zhang Y.-F., Sun J.-T., Zhang Y.-Y., Du S. (2021). Database Construction for Two-Dimensional Material-Substrate Interfaces. Chin. Phys. Lett..

[12] He S., Chen S., Zhao Y., Qi N., Zhan X. (2021). Study on the intelligent model database modeling the laser welding for aerospace aluminum alloy. J. Manuf. Process..

[13] Coudert F. (2019). Materials Databases: The Need for Open, Interoperable Databases with Standardized Data and Rich Metadata. Adv. Theory Simul..

[14] Jia D., Duan H., Zhan S., Jin Y., Cheng B., Li J. (2019). Design and development of lubricating material database and research on performance prediction method of machine learning. Sci. Rep..

[15] Surmiak M.A., Zhang T., Lu J., Rietwyk K.J., Raga S.R., McMeekin D.P., Bach U. (2020). High-Throughput Characterization of Perovskite Solar Cells for Rapid Combinatorial Screening. Sol. RRL.

[16] Li Z., Xu Q., Sun Q., Hou Z., Yin W.-J. (2019). Thermodynamic stability landscape of halide double perovskites via high-throughput computing and machine learning. Adv. Funct. Mater..

[17] Yang K., Xu X., Yang B., Cook B., Ramos H., Krishnan N.M.A., Smedskjaer M.M., Hoover C., Bauchy M. (2019). Predicting the young’s modulus of silicate glasses using high-throughput molecular dynamics simulations and machine learning. Sci. Rep..

[18] Li X., Yang X., Liu L., Zhou P., Zhou J., Shi X., Wang Y. (2020). A microarray platform designed for high-throughput screening the reaction conditions for the synthesis of micro/nanosized biomedical materials. Bioact. Mater..

[19] Coury F.G., Wilson P., Clarke K.D., Kaufman M.J., Clarke A.J. (2019). High-throughput solid solution strengthening characterization in high entropy alloys. Acta Mater..

[20] Wang J., Xiao B., Liu Y. (2020). Machine learning assisted high-throughput experiments accelerates the composition design of hard high-entropy alloy coxcrytizmouwv. Mater. China.

[21] Kheiri S., Mohamed M.G., Amereh M., Roberts D., Kim K. (2020). Antibacterial efficiency assessment of polymer-nanoparticle composites using a high-throughput microfluidic platform. Mater. Sci. Eng. C.

[22] Yuan W., Zhang L., Tao G., Wang S., Wang Y., Zhu Q., Zhang G., Zhang Z., Xue Y., Qin S. (2020). Designing high-performance hypergolic propellants based on materials genome. Sci. Adv..

[23] Esmaeilzadeh-Gharedaghi E., Faramarzi M.A., Amini M.A., Najafabadi A.R., Rezayat S.M., Amani A. (2012). Effects of processing parameters on particle size of ultrasound prepared chitosan nanoparticles: An artificial neural networks study. Pharm. Dev. Technol..

[24] Wang S., Wang S., Wu H.-H., Wu Y., Mi Z., Mao X. (2021). Towards enhanced strength-ductility synergy via hierarchical design in steels: From the material genome perspective. Sci. Bull..

[25] Liang Y. (2021). Exploring inorganic and nontoxic double perovskites Cs_2_AgInBr_6(1−x)_Cl_6x_ from material selection to device design in material genome approach. J. Alloys Compd..

[26] Ren F., Ward L., Williams T., Laws K.J., Wolverton C., Hattrick-Simpers J., Mehta A. (2018). Accelerated discovery of metallic glasses through iteration of machine learning and high-throughput experiments. Sci. Adv..

[27] Huang Y., Zhang T., Zhu Z., Gong W., Xia X. (2021). PM2.5 concentration estimation with 1-km resolution at high coverage over urban agglomerations in China using the BPNN-KED approach and potential application. Atmos. Res..

[28] Bai B., Zhang J., Wu X., Zhu G., Li X. (2021). Reliability prediction-based improved dynamic weight particle swarm optimization and back propagation neural network in engineering systems. Expert Syst. Appl..

[29] Han X., Wei Z., Zhang B., Li Y., Du T., Chen H. (2021). Crop evapotranspiration prediction by considering dynamic change of crop coefficient and the precipitation effect in back-propagation neural network model. J. Hydrol..

[30] Chen Y., Mao Y., Pan X., Jin W., Qiu T. (2021). Verification and comparison of three prediction models of ischemic stroke in young adults based on the back propagation neural networks. Medicine.

[31] Qiu W., Wen G., Liu H. (2021). A back-propagation neural network model based on genetic algorithm for prediction of build-up rate in drilling process. Arab. J. Sci. Eng..

[32] Spoorani S., Balasubramanie P. (2021). Seizure Detection Based on EEG Signals Using Asymmetrical Back Propagation Neural Network Method. Circuits, Syst. Signal Process..

[33] Du B., Lund P.D., Wang J., Kolhe M., Hu E. (2021). Comparative study of modelling the thermal efficiency of a novel straight through evacuated tube collector with mlr, svr, bp and rbf methods. Sustain. Energy Technol. Assess..

[34] Cai A., Xiong X., Liu Y., An W., Zhou G., Luo Y., Li T., Li X., Tan X. (2014). Compositional optimization of glass forming alloys based on critical dimension by using artificial neural network. Trans. Nonferrous Met. Soc. China.

[35] Zacharia K., Krishnakumar P. (2020). Chatter Prediction in High Speed Machining of Titanium Alloy (Ti-6Al-4V) using Machine Learning Techniques. Mater. Today: Proc..

[36] Hashad R.A., Ishak R.A.H., Fahmy S., Mansour S., Geneidi A.S. (2016). Chitosan-tripolyphosphate nanoparticles: Optimization of formulation parameters for improving process yield at a novel ph using artificial neural networks. Int. J. Biol. Macromol..

[37] Siafaka P.I., Barmpalexis P., Lazaridou M., Papageorgiou G.Z., Koutris E., Karavas E., Kostoglou M., Bikiaris D.N. (2015). Controlled release formulations of risperidone antipsychotic drug in novel aliphatic polyester carriers: Data analysis and modelling. Eur. J. Pharm. Biopharm..

[38] Soltani S., Shojaei T.R., Khanian N., Choong T.S.Y., Asim N., Rashid U. (2021). Porosity Estimation of Mesoporous TiO_2_-ZnO Nanocrystalline by Artificial Neural Network Modeling. Chem. Eng. Technol..

[39] Rojek I., Mikołajewski D., Dostatni E., Macko M. (2020). Ai-optimized technological aspects of the material used in 3d printing processes for selected medical applications. Materials.

[40] Wu H., Zhao Y., Tan H. (2021). Novel radial basis function network based on dynamic time warping and kalman filter for real-time monitoring of supersonic inlet flow patterns. J. Aerosp. Eng..

[41] Lv Z., Xiao F., Wu Z., Liu Z., Wang Y. (2021). Hand gestures recognition from surface electromyogram signal based on self-organizing mapping and radial basis function network. Biomed. Signal Process. Control..

[42] Liu Q., Li D., Ge S.S., Ji R., Ouyang Z., Tee K.P. (2021). Adaptive bias rbf neural network control for a robotic manipulator. Neurocomputing.

[43] Fang Q. (2021). Estimation of navigation mark floating based on fractional-order gradient descent with momentum for rbf neural network. Math. Probl. Eng..

[44] Vives-Boix V., Ruiz-Fernández D. (2021). Fundamentals of artificial metaplasticity in radial basis function networks for breast cancer classification. Neural Comput. Appl..

[45] Shang D., Li X., Yin M., Li F. (2021). Control Method of Flexible Manipulator Servo System Based on a Combination of RBF Neural Network and Pole Placement Strategy. Mathematics.

[46] Han H., Ma M., Qiao J. (2021). Accelerated gradient algorithm for rbf neural network. Neurocomputing.

[47] Zhang H., Xiong L., Xu Z., Liu X. (2017). Preparation of double-shell phase change and humidity storage micro-capsules with uniform particle size distribution. Acta Mater. Compos. Sin..

[48] Duriagina Z., Tkachenko R., Trostianchyn A., Lemishka I., Kovalchuk A., Kulyk V., Kovbasyuk T. (2018). Determination of the best microstructure and titanium alloy powders properties using neural network. J. Achiev. Mater. Manuf. Eng..

[49] Wang Y. (2020). An Fault Diagnosis Method for Planetary Gear Based on Differential Evolution for Probabilistic Neural Network. Master’s Thesis.

[50] He X. (2018). Classification of Arrhythmias Based on the Ga-Pnn Model. Master’s Thesis.

[51] Xiong L. (2019). Research on Recognition of Chd Heart Sound Based on Wavelet Cepstrum Coefficient and Probabilistic Neural Network. Master’s Thesis.

[52] Chen S. (2018). The Study of Graph Matching Based on Probabilistic Neural Network. Master’s Thesis.

[53] Zhuang Q. (2017). Probabilistic Neural Network on Image Emotion Classification. Master’s Thesis.

[54] Sun Y.T., Bai H.Y., Li M.Z., Wang W.H. (2017). Machine Learning Approach for Prediction and Understanding of Glass-Forming Ability. J. Phys. Chem. Lett..

[55] Izonin I., Trostianchyn A., Duriagina Z., Tkachenko R., Tepla T., Lotoshynska N. (2018). The Combined Use of the Wiener Polynomial and SVM for Material Classification Task in Medical Implants Production. Int. J. Intell. Syst. Appl..

[56] Liu D., Zheng P., Cao M., Yin H., Xu Y., Zhang L. (2021). A new method of roundness error evaluation based on twin support vector machines. Meas. Sci. Technol..

[57] Jueyendah S., Lezgy-Nazargah M., Eskandari-Naddaf H., Emamian S. (2021). Predicting the mechanical properties of cement mortar using the support vector machine approach. Constr. Build. Mater..

[58] Zhang W., Xie P., Li Y., Zhu J. (2021). A machine learning model for predicting the mass transfer performance of rotating packed beds based on a least squares support vector machine approach. Chem. Eng. Process.—Process Intensif..

[59] Yin C., Deng X., Yu Z., Chen R., Zhong H., Liu Z., Cai G., Zheng Q., Liu X., Zhong J. (2021). Auto-classification of biomass through characterization of their pyrolysis behaviors using thermogravimetric analysis with support vector machine algorithm: Case study for tobacco. Biotechnol. Biofuels.

[60] Meher P.K., Mohapatra A., Satpathy S., Sharma A., Saini I., Pradhan S.K., Rai A. (2021). PredCRG: A computational method for recognition of plant circadian genes by employing support vector machine with Laplace kernel. Plant Methods.

[61] Ganesan K., Sureshbabu M. (2021). Deep proximal support vector machine classifiers for hyperspectral images classification. Neural Comput. Appl..

[62] Zhu Q., Wang Y., Luo Y. (2021). Improvement of multi-layer soil moisture prediction using support vector machines and ensemble kalman filter coupled with remote sensing soil moisture datasets over an agriculture dominant basin in China. Hydrol. Process..

[63] Moslemnejad S., Hamidzadeh J. (2021). Weighted support vector machine using fuzzy rough set theory. Soft Comput..

[64] Guo Y., Zhang Z., Tang F. (2021). Feature selection with kernelized multi-class support vector machine. Pattern Recognit..

[65] Zhang L., Chen H., Tao X., Cai H., Liu J., Ouyang Y., Peng Q., Du Y. (2020). Machine learning reveals the importance of the formation enthalpy and atom-size difference in forming phases of high entropy alloys. Mater. Des..

[66] Tkachenko R., Duriagina Z., Lemishka I., Izonin I., Trostianchyn A. (2018). Development of machine learning method of titanium alloy properties identification in additive technologies. East.-Eur. J. Enterp. Technol..

[67] Liu J., Dong Z., Xia J., Wang H., Meng T., Zhang R., Han J., Wang N., Xie J. (2021). Estimation of soil organic matter content based on cars algorithm coupled with random forest. Spectrochim. Acta Part A: Mol. Biomol. Spectrosc..

[68] Abellán-García J., Guzmán-Guzmán J.S. (2021). Random forest-based optimization of uhpfrc under ductility requirements for seismic retrofitting applications. Constr. Build. Mater..

[69] Lin Z., Lin S., Neamtiu I.A., Ye B., Csobod E., Fazakas E., Gurzau E. (2021). Predicting environmental risk factors in relation to health outcomes among school children from romania using random forest model—An analysis of data from the sinphonie project. Sci. Total Environ..

[70] Wolfensberger D., Gabella M., Boscacci M., Germann U., Berne A. (2021). RainForest: A random forest algorithm for quantitative precipitation estimation over Switzerland. Atmos. Meas. Tech..

[71] Wang Z., Zhang X., Chhin S., Zhang J., Duan A. (2021). Disentangling the effects of stand and climatic variables on forest productivity of Chinese fir plantations in subtropical China using a random forest algorithm. Agric. For. Meteorol..

[72] Speiser J.L. (2021). A random forest method with feature selection for developing medical prediction models with clustered and longitudinal data. J. Biomed. Informatics.

[73] Li M., Xu Y., Men J., Yan C., Tang H., Zhang T., Li H. (2021). Hybrid variable selection strategy coupled with random forest (rf) for quantitative analysis of methanol in methanol-gasoline via raman spectroscopy. Spectrochim. Acta Part A Mol. Biomol. Spectrosc..

[74] Li X., Cao Z., Dan Y., Niu C., Hu J. (2019). Prediction of metal oxide performance based on machine learning and multi-scale feature. N. Chem. Mater..

[75] Fan Y., Bai J., Lei X., Lin W., Hu Q., Wu G., Guo J., Tan G. (2021). Ppmck: Privacy-preserving multi-party computing for k-means clustering. J. Parallel Distrib. Comput..

[76] Zukotynski K.M., Black S.E.D., Kuo P.H.M., Bhan A.M., Adamo S.H., Scott C.J.M., Lam B., Masellis M.M., Kumar S., Fischer C.E. (2021). Exploratory Assessment of K-means Clustering to Classify 18F-Flutemetamol Brain PET as Positive or Negative. Clin. Nucl. Med..

[77] Rim B., Lee S., Lee A., Gil H.-W., Hong M. (2021). Semantic cardiac segmentation in chest ct images using k-means clustering and the mathematical morphology method. Sensors.

[78] Liu B., Zhang T., Li Y., Liu Z., Zhang Z. (2021). Kernel probabilistic k-means clustering. Sensors.

[79] Bae J., Kim M., Lim J., Geem Z. (2021). Feature Selection for Colon Cancer Detection Using K-Means Clustering and Modified Harmony Search Algorithm. Mathematics.

[80] Ni T., Qiao M., Chen Z., Zhang S., Zhong H. (2021). Utility-efficient differentially private k-means clustering based on cluster merging. Neurocomputing.

[81] Xiong P., Liu H., Tian Y., Chen Z., Wang B., Yang H. (2020). Helicopter maritime search area planning based on a minimum bounding rectangle and K-means clustering. Chin. J. Aeronaut..

[82] Li M.-A., Ruan Z. (2021). A novel decoding method for motor imagery tasks with 4d data representation and 3d convolutional neural networks. J. Neural Eng..

[83] Mishra P., Passos D. (2021). Deep multiblock predictive modelling using parallel input convolutional neural networks. Anal. Chim. Acta.

[84] Liz H., Sánchez-Montañés M., Tagarro A., Domínguez-Rodríguez S., Dagan R., Camacho D. (2021). Ensembles of Convolutional Neural Network models for pediatric pneumonia diagnosis. Futur. Gener. Comput. Syst..

[85] Carloto I., Johnston P., Pestana C.J., Lawton L.A. (2021). Detection of morphological changes caused by chemical stress in the cyanobacterium Planktothrix agardhii using convolutional neural networks. Sci. Total. Environ..

[86] Zhang P., Yin Z.-Y. (2021). A novel deep learning-based modelling strategy from image of particles to mechanical properties for granular materials with CNN and BiLSTM. Comput. Methods Appl. Mech. Eng..

[87] Jia S., Hu P. (2021). Chrnet: A re-trainable chromosome-based 1d convolutional neural network for predicting immune cell types. Genomics.

[88] Kan X., Fan Y., Fang Z., Cao L., Xiong N.N., Yang D., Li X. (2021). A novel iot network intrusion detection approach based on adaptive particle swarm optimization convolutional neural network. Inf. Sci..

[89] Mcclelland J., Rumelhart D., Hinton G. (1988). The Appeal of Parallel Distributed Processing. Readings in Cognitive Science.

[90] Broomhead D.S., Lowe D. (1988). Radial Basis Functions, Multi-Variable Functional Interpolation and Adaptive Networks, No. RSRE-MEMO-4148. Royal Signals and Radar Establishment Malvern (United Kingdom).

[91] Specht D.F. (1990). Probabilistic neural networks. Neural Netw..

[92] Cortes C., Vapnik V. (1995). Support-vector networks. Mach. Learn..

[93] Drucker H., Burges C.C., Kaufman L., Smola A.J., Vapnik V.N.A. Support vector regression machines. Proceedings of the Advances in Neural Information Processing Systems 9.

[94] Ho T.K. Random Decision Forests. Proceedings of the 3rd International Conference on Document Analysis and Recognition.

[95] Pelleg D., Moore A. Accelerating exact k-means algorithms with geometric reasoning. Proceedings of the Fifth ACM SIGKDD International Conference on Knowledge Discovery and Data Mining.

[96] Homma T., Atlas L.E., Marks R.J. (1987). An artificial neural network for spatio-temporal bipolar patterns: Application to phoneme classification. Neural Information Processing Systems 0 (NIPS 1987).

[97] Hao Y. (2019). The Construction of Microstructure on Biomedical Metallic Materials and Their Biological Functional Evaluation. Master’s Thesis.

[98] Zhang W. (2020). Research status and application progress of biomedical metal materials. Met. World.

[99] Zhao X., Wang C., Liu H., Ma X., Cheng J., Yu S., Yu Z. (2018). Research progress and application of first principle calculation in novel bio-metal materials. Mater. Rev..

[100] Wang Q., Ji Y., Xu D.-K. (2019). Research progress on the corrosion fatigue of biomedical metallic alloys. Surf. Technol..

[101] Johnson W.L. (1999). Bulk glass-forming metallic alloys: Science and technology. MRS Bull..

[102] Wang X., Chen X., Xia T., Kang K., Peng B. (2006). The current situation of amorphous alloy application. Mater. Rev..

[103] Schroers J. (2010). Processing of Bulk Metallic Glass. Adv. Mater..

[104] Li M.-X. (2019). Combinatorial Method and Thermal Training on Metallic Glasses. Ph.D. Thesis.

[105] Maldonis J.J., Banadaki A.D., Patala S., Voyles P.M. (2019). Short-range order structure motifs learned from an atomistic model of a zr50cu45al5 metallic glass. Acta Mater..

[106] Zhang Y., Liu H., Wang C., Cheng J., Shi J., Wang L., Yu Z. (2017). Development trend and research application situation of biomedical metal materials. Hot Work. Technol..

[107] Ren J., Zhang Y., Tan J., Sui Y., Wang J., Zhang X. (2016). Current research status and trend of titanium alloys for biomedical applications. Mater. Rev..

[108] Zhang J., Li X., Xu D., Yang R. (2019). Recent progress in the simulation of microstructure evolution in titanium alloys. Prog. Nat. Sci..

[109] Banerjee R., Nag S., Fraser H.L. (2005). A novel combinatorial approach to the development of beta titanium alloys for orthopaedic implants. Mater. Sci. Eng. C.

[110] Wan P., Wang K., Lu S., Chen X., Zhou F. (2019). Constitutive modeling of ti-2.7cu alloy based on strain compensation and pso-bp neural network. J. Mater. Eng..

[111] Tepla T.L., Izonin I.V., Duriagina Z.A., Tkachenko R.O., Trostianchyn A.M., Lemishka I.A., Kulyk V.V., Kovbasyuk T.M. (2018). Alloys selection based on the supervisedlearning technique for design ofbiocompatible medical materials. Arch. Mater. Sci. Eng..

[112] Hu S. (2019). Study on Microstructure and Properties of Biomedical Titanium Type High Entropy Alloy. Master’s Thesis.

[113] Moorehead M., Bertsch K., Niezgoda M., Parkin C., Elbakhshwan M., Sridharan K., Zhang C., Thoma D., Couet A. (2020). High-throughput synthesis of Mo-Nb-Ta-W high-entropy alloys via additive manufacturing. Mater. Des..

[114] Choi Y., Jang J., Koo H.-J., Tanaka M., Lee K.-H., Choi J. (2021). Alginate-chitosan hydrogel patch with beta-glucan nanoemulsion for antibacterial applications. Biotechnol. Bioprocess Eng..

[115] Shi J. (2020). Application of biomedical polymer materials in medical treatment. Mod. Chem. Res..

[116] Baharifar H., Amani A. (2016). Size, Loading Efficiency, and Cytotoxicity of Albumin-Loaded Chitosan Nanoparticles: An Artificial Neural Networks Study. J. Pharm. Sci..

[117] Louzao I., Koch B., Taresco V., Cantu L.A.R., Irvine D.J., Roberts C.J., Tuck C., Alexander C., Hague R., Wildman R. (2018). Identification of Novel “Inks” for 3D Printing Using High-Throughput Screening: Bioresorbable Photocurable Polymers for Controlled Drug Delivery. ACS Appl. Mater. Interfaces.

[118] Kim C., Chandrasekaran A., Huan T.D., Das D., Ramprasad R. (2018). Polymer genome: A data-powered polymer informatics platform for property predictions. J. Phys. Chem. C.

[119] Mikulskis P., Hook A.L., Dundas A.A., Irvine D.J., Sanni O., Anderson D.G., Langer R., Alexander M.R., Williams P., Winkler D.A. (2018). Prediction of Broad-Spectrum Pathogen Attachment to Coating Materials for Biomedical Devices. ACS Appl. Mater. Interfaces.

[120] Li Y., Chen K. (2018). Research progress of poly(lactic acid) biomedical composite materials. Shandong Chem. Ind..

[121] Yang W., Zhang C., Ma C. (2017). Thickness prediction of micro-arc oxidation coating on magnesium alloy based on ga-bp neural network. Ordnance Mater. Sci. Eng..

[122] Mou P. (2018). Fabrication of Gradient tio2 Nanotubes for High-Throughput Screening of Biological Responses. Master’s Thesis.

[123] Jahan K., Tabrizian M. (2016). Composite biopolymers for bone regeneration enhancement in bony defects. Biomater. Sci..

[124] Sun P. (2019). Preparation and Properties of Polylatic Acid-Based Biomedical Composites. Master’s Thesis.

[125] Seyedhassantehrani N., Karimi R., Tavoosidana G., Amani A. (2016). Concurrent study of stability and cytotoxicity of a novel nanoemulsion system—An artificial neural networks approach. Pharm. Dev. Technol..

[126] Zaki M.R., Varshosaz J., Fathi M. (2015). Preparation of agar nanospheres: Comparison of response surface and artificial neural network modeling by a genetic algorithm approach. Carbohydr. Polym..

[127] Li Y., Abbaspour M.R., Grootendorst P.V., Rauth A.M., Wu X.Y. (2015). Optimization of controlled release nanoparticle formulation of verapamil hydrochloride using artificial neural networks with genetic algorithm and response surface methodology. Eur. J. Pharm. Biopharm..

[128] Zhang J., Hu X., Li G., Ju X., Chu L., Wang Y. (2019). Applications of marine-derived chitosan and alginates in biemedicine. J. Biomed. Eng..

[129] Soltanzadeh M., Peighambardoust S.H., Ghanbarzadeh B., Mohammadi M., Lorenzo J.M. (2021). Chitosan nanoparticles as a promising nanomaterial for encapsulation of pomegranate (*Punica granatum* L.) Peel extract as a natural source of antioxidants. Nanomaterials.

[130] Shang X. (2016). Preparation and Performance Research of Collagen/Chitosan Composite Microspheres and Membrane. Master’s Thesis.

[131] Wu X., Gao J., Ding P. (2022). Ensemble learning of polypropylene-composite aging data. J. Shanghai Univ. (Nat. Sci. Ed.).

[132] Liu F., Fan H., Lü T., Li Q., Qian Q. (2022). Kalman filter based mathod for processing small noisy sample data. J. Shanghai Univ. (Nat. Sci. Ed.).

[133] Wu X., Hu M., Ding P. (2022). Multi-modal data representation learning for ceramic coating materials. J. Shanghai Univ. (Nat. Sci. Ed.).

